# Costs and difficulties of recruiting patients to provide e-health support: pilot study in one primary care trust

**DOI:** 10.1186/1472-6947-12-25

**Published:** 2012-03-29

**Authors:** Ray B Jones, Anita O'Connor, Jade Brelsford, Neil Parsons, Heather Skirton

**Affiliations:** 1 Faculty of Health, Education and Society, Plymouth University, Plymouth PL4 8AA, UK; 2 NHS Plymouth, Building Two, Derriford Business Park, Derriford Plymouth PL6 5QZ, UK; 3 Sentinel GP Community Interest Company, Building Two, Derriford Business Park, Derriford Plymouth PL6 5QZ, UK

**Keywords:** Recruitment strategies, General practice, Long term conditions, Digital divide, Email support, Pilot study

## Abstract

**Background:**

Better use of e-health services by patients could improve outcomes and reduce costs but there are concerns about inequalities of access. Previous research in outpatients suggested that anonymous personal email support may help patients with long term conditions to use e-health, but recruiting earlier in their 'journey' may benefit patients more. This pilot study explored the feasibility and cost of recruiting patients for an e-health intervention in one primary care trust.

**Methods:**

The sample comprised 46 practices with total patient population of 250,000. We approached all practices using various methods, seeking collaboration to recruit patients via methods agreed with each practice. A detailed research diary was kept of time spent recruiting practices and patients. Researcher time was used to estimate costs. Patients who consented to participate were offered email support for their use of the Internet for health.

**Results:**

Eighteen practices agreed to take part; we recruited 27 patients, most (23/27) from five practices. Practices agreed to recruit patients for an e-health intervention via waiting room leaflets (16), posters (16), practice nurses (15), doctors giving patients leaflets (5), a study website link (7), inclusion in planned mailshots (2), and a special mailshot to patients selected from practice computers (1). After low recruitment response we also recruited directly in five practices through research assistants giving leaflets to patients in waiting rooms. Ten practices recruited no patients. Those practices that were more difficult to recruit were less likely to recruit patients. Leaving leaflets for practice staff to distribute and placing posters in the practice were not effective in recruiting patients. Leaflets handed out by practice nurses and website links were more successful. The practice with lowest costs per patient recruited (£70) used a special mailshot to selected patients.

**Conclusion:**

Recruitment via general practice was not successful and was therefore expensive. Direct to consumer methods and recruitment of patients in outpatients to offer email support may be more cost effective. If recruitment in general practice is required, contacting practices by letter and email, not following up non-responding practices, and recruiting patients with selected conditions by special mailshot may be the most cost-effective approach.

## Background

E-health services include: (i) seeking information from online resources, (ii) interacting with an application that aims to support patient decision making or change health-related behaviour, (iii) viewing or contributing to their medical records, (iv) seeking emotional or information support from their peers, or (v) communicating with professionals online. Although not consistent across this range, there is increasing evidence that people with long term conditions using e-health services can better manage their care, thus achieving better health outcomes, than those that do not use e-health [1].

However, there are continuing concerns over e-health inequalities [2,3] and steps taken to try to counter them [4]. Although e-health inequalities may also result from lack of physical access or for economic reasons, e-health literacy [3,5-10] is one component for which improved support for patients may have positive effects. The types of support that prove effective will vary across the spectrum of e-health literacy. For example, young frequent Internet and social network users may benefit from a better understanding of privacy, confidentiality and security issues for e-health. Older people who have yet to start using the Internet may require convincing of the benefit and face-to-face instruction on getting started. This study focused on those people who already had Internet access but may have lacked confidence to use the full range of possibilities for e-health.

The problems of recruiting patients to studies, particularly in general practice are well known. Campbell et al. reviewed 114 randomised trials noting the problems of recruitment and recommending further research into different recruitment patterns including 'failures' [11]. Murray et al. have reviewed the special problems encountered in online recruitment [12], noting that online recruitment even more than traditional methods can potentially result in an unrepresentative sample.

Recruiting participants for studies of Internet based interventions is increasingly common (for example [13]). Usual practice is to have a registration website and to use various means including online advertisements [14-16], emails to relevant groups [17], as well as 'offline' methods such as press, letters, posters, TV, and radio [15,18] to raise awareness and encourage potential participants to visit. Online methods seem more cost-effective in recruiting for online interventions. However, it was difficult to know the best way of raising awareness for our target group given its special characteristics. As the aim is to encourage use of the Internet by more naïve users or people who would not have thought of using it for health, raising awareness 'offline' for online recruitment may be more appropriate.

In a previous study, we piloted email support for people with long term conditions who were less experienced users of the Internet, by raising awareness of the study in outpatient clinics in an acute hospital [17]. We recruited 39 patients. Most were made aware of the study by a researcher distributing leaflets in outpatient waiting areas. Patients were willing to correspond by email with an e-health facilitator. However, some participants suggested that recruiting patients earlier in 'their journey', in primary care would be more beneficial. Also, recruiting in an acute hospital meant that we were unable to make use of the full range of effective Internet interventions, such as those addressing mild to moderate mental health issues or lifestyle changes, which are more appropriate for general practice. This study explored the feasibility and cost of recruiting patients in general practice for email support for e-health.

Staff in general practice have the opportunity to use practice systems to identify patients who may benefit from Internet use. For example, by searching for people with the diagnosis of depression [19,20], or all those who smoke, or with a particular long term condition, patients can be recommended to use an e-health service [21]. Such methods have been used before with variable success for depression [19,20,22]. However, it was not known if general practitioners (GPs) were willing to engage in the promotion of e-health support in this way, nor what methods were acceptable to them and their patients in raising awareness of e-health.

In order to compare recruitment via general practice with secondary care, or direct to population methods such as mass media campaigns or online advertising [15,23,24], it is essential to determine how much it might cost to recruit patients to a study that offers them supported access to online health information. A literature search failed to identify any previous studies reporting costs of recruiting patients for this type of intervention. Therefore, the aim of this pilot study was to explore the feasibility and cost of recruiting patients to email support for e-health in general practice. Research questions were: (i) which methods of contacting and recruiting practices seem effective, (ii) how much does it cost to recruit practices, (iii) what methods of patient recruitment are practices willing to use, (iv) how many patients are recruited via different methods, (v) what is the cost per patient recruited?

## Methods

### Ethics

The study was approved by the South West National Health Service Ethics Committee.

### Overall design

This was a phase one pilot study [25] using mixed methods to test the feasibility of recruitment to an intervention to support people's use of the Internet for health. The study ran from January to July 2011.

### Setting

In 2011, England organised primary care services through 152 primary care trusts. We used a convenience sample of one primary care trust, Plymouth, in Southwest England with 46 practices serving a population of 250,000. We aimed to contact and gain collaboration from as many practices as possible in this trust and, by using methods agreed with practices, to recruit patients to the study with the offer of email support in their use of e-health. Participants were to be directed towards a relevant part of the NHS Choices website http://www.nhs.uk and asked about what help or support they needed by email dialogue.

### Research diary

A diary was kept by the research team of all contacts with practices, time spent on different activities (to allow costing), and comments made by professionals and patients.

### Recruitment of practices and agreement on their level of participation

Practices were contacted by letter, phone, email, or personal visit to find out if they would collaborate in the recruitment of patients and what form that recruitment might take. Methods of patient recruitment suggested to practices included 'passive' methods of raising awareness (posters/leaflets available in the practice, and links on their websites), and more proactive methods such as GPs or practice nurses giving patients leaflets, or practice staff using their computer systems to identify relevant cohorts of patients to mail invitations to join the study. Suggested cohorts included those with long term conditions such as heart disease, depression, and asthma, or an aspect of their life they would like to change such as losing weight or giving up smoking. To control costs, we initially emphasised to practice managers the option of including project information in previously planned practice mailshots, with the option of a special mailshot to patients (with costs paid for by the project) receiving less emphasis. We were led by the practice manager in their choice of methods that seemed to fit best with their practice.

### Recruitment of patients

Patients were given a leaflet or saw a poster or a website link about the project, inviting them to visit the project website where they would register for the project using the reference number from that source. How patients received this leaflet depended on the participation chosen by the practice. The website included more information about the study, asked for the code number of the leaflet, letter, or other notification, allowing us to know how they received notification of the study. The website also asked for their GP practice, age range, health condition for which they were joining the study, and invited them to consent by giving their email address. They were subsequently asked to complete an online questionnaire that asked for gender, occupation, and their current use of the Internet.

### Email support

Recruited patients were contacted by a researcher (JB) and offered help in using the Internet. This study told us nothing new compared to our previous project [17]. As this paper focusses on recruitment, we have not reported the email correspondence further.

### Cost analysis

We estimated costs for different forms of patient recruitment based on marginal time costs for research assistants and practice staff and consumable costs. Researcher time, recorded in the research diary for all activities, was divided between research (needed for this study only) and service (if patients were being routinely recruited for email support), fixed (per project) and variable (per practice) costs. Examples are given in Table 1.

**Table 1:** Examples of classification of activities to cost type

Research	Fixed	Creating study spreadsheet, writing interim report, preparing for meeting, meeting with research team, ethical approval, project website etc.
	
	Per practice	Writing up practice notes on contacts and current status, writing up practice visit notes, etc.
**Service**	Fixed	Designing and having advertising materials printed, arranging and attending meeting with practice nursing group.
	
	Per practice	Phone calls and emails to practices, personalising letters to be sent to practices, delivering leaflets to practices, visits to practices to explain recruitment, maintaining record of practice status.

For completeness, we documented research activities, but in this analysis examine only 'service costs'. In addition to the cost of researcher time there was the cost of practice time. We asked practices to keep a note of time spent on the project. Four practices gave estimates and we estimated practice time based on the mean of these four estimates. Researcher and practice time was costed at £20/hour, based on a typical hourly researcher rate including oncosts; researchers on a salary of £22931 cost the University £29305 marginal cost for a 1650 hour year. We rounded up the hourly cost of £17.76 to £20 to allow for (marginal) cost supervision of the researchers. We did not include overhead or estate costs. We also included: printing costs (leaflets, posters, letters), and postage costs (letters to practices), and the cost paid to one practice for a special mailshot (£200). We did not include patient time/costs in using the website to consent.

The total (variable service) practice cost was therefore the sum of:

• Printing and postage costs (all practices initial letter)

• Total researcher time at £20/hour recruiting and supporting each practice

• Total practice time (estimated at 2 hours) at £20/hour for participating practices

• Total printing costs for leaflets and posters for each practice.

We did not include telephone call charges, time spent by practices that did not participate, or mileage costs in visiting practices.

### Cost reimbursement to practices

Practices were not offered any reimbursement for their involvement in the study as the offer of email support should have been a benefit for their patients. Although we asked practices to document time spent on the project (as above), costs were likely to be largely 'hidden costs'. The one exception was the cost of a 'special mailshot' to identify patients which would involve practice staff interrogating the practice computer with subsequent printing and postage costs for the mailshot. One practice offered to take this approach and we agreed reimbursement of £1/patient for 200 patients.

### Leaflets distributed by research assistants in general practice waiting rooms

When recruitment of patients proved very slow we introduced a new method of recruitment, namely that researchers would distribute leaflets to patients in the waiting room. Because of the project's limited resources this was proposed only to the five practices that had shown most enthusiasm for the project. Prior to the researchers' visits, consent was obtained from each of the practice managers and a time and date agreed. Each practice offered different visit times including during special clinics for chronic disease management, anti-coagulant checks, diabetes, asthma, as well as during general surgery times.

## Results

### Numbers of practices participating

**Figure 1:**
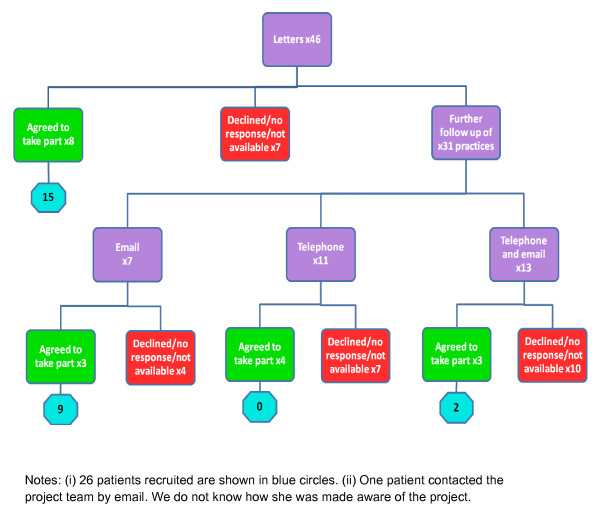
**Methods of contact, practice responses to recruitment, and subsequent number of patients recruited**.

Figure 1 shows the method of recruitment of practices. Eight out of 46 practices responded positively to the initial letter. Thirty-one were followed up with further contacts. Initially we only had email addresses for seven practices so most (24) were contacted by trying to telephone the practice. Obtaining access to speak to the practice manager was time consuming requiring repeated attempts. Of those contacted some (13) then gave an email address and asked for further details so that acceptance or rejection of participation came after different forms of contact. In total 18/46 (39%) practices agree to participate in the study. Levels of deprivation are higher in Plymouth than in England as a whole [26] but the 18 practices were distributed throughout the primary care trust including deprived and more affluent areas.

### What were practices prepared to do?

Of the 18 practices, 16 agreed to have leaflets in the waiting room, 16 had posters, 15 agreed for practice nurses to give selected patients leaflets. Ten said they would add a website link on the website (but only seven did). Six agreed to identify patients from their practice register and send out letters along with previously arranged mailshots (but only one practice did, to 20-30 Chronic Obstructive Pulmonary Disease and Ischaemic Heart Disease patients). One practice agreed to send out letters to 200 patients with depression if reimbursed £200 for administrative costs. Table 2 shows the combinations of these methods. In total, we distributed 2,190 leaflets and 44 posters to the 18 practices.

**Table 2:** Numbers of patients recruited by different methods

	Methods of patient recruitment used by practice						
	**Leaflets only**	**Leaflets and posters**	**Leaflets, posters, and weblinks**	**Leaflets, posters, and integrated mailshot**	**Leaflets, posters, weblinks, and research assistant**	**Leaflets, posters, weblinks, research assistants, and special mailshot**	**Total**

Number of practices that recruited patients	2	8	2	1	4	1	**18**

Number of patients recruited	0	1	1	2	16	7	**27**

Patient recruitment method as determined by registration code							

Posters		0	0	0	0	0	

Leaflets given by a practice nurse		0	0	0	6	0	**6**

Leaflets given by a receptionist		-	-	-	-	1	**1**

Leaflets given by a doctor		0	-	0	0	-	**0**

Leaflets available in the waiting room		1	1	1	2	0	**5**

Leaflets given by a research assistant in the waiting room		-	-	-	2	0	**2**

Weblink		-	0	-	5	0	**5**

Integrated mailshot		-	-	1	-	-	**1**

Special mailshot		-	-	-	-	6	**6**

Email to researcher		-	-	-	1	-	**1**

### Numbers of patients recruited by different methods

Although 18 practices participated, we only recruited 27 patients in eight practices; five practices were responsible for 23/27 patients. As well as knowing which practices recruited patients we know from code numbers given by patients on leaflets or other materials which method was responsible for their recruitment. Table 2 shows the numbers of patients recruited by different methods compared to the methods used by practices (one patient emailed the team to ask to be included). The practice that carried out the special mailshot for a charge of £200 recruited seven patients, six via the special mailshot. Although those practices which had leaflets distributed by the research assistant recruited more patients than others at a lower cost, only two were apparently recruited as a direct result of being given leaflets by the research assistant. Six patients were recruited in two practices by practice nurses and five were recruited by a website link. Three other practices did not recruit anyone by a website link.

### Characteristics of patients recruited

Sixteen of the 27 had a long term condition including depression, asthma, psoriasis, urinary problems, osteoarthritis, diabetes, fibromyalgia, atrial fibrillation, amputation and congenital heart defect. Five people wanted to lose weight. The median age was 55, with 4 patients being over 60 and 3 over 70. Twenty-three answered the online questionnaire; 13 were women. Ten participants were in paid employment, seven retired, three long term sick, and three were housewives. All but one said they used the Internet for browsing and email, four had used discussion forums, six had used Skype, but 13/23 had used social network sites. Ten had used their GP practice website but 15/23 said they had not used the Internet for their long term condition. We did not specifically ask, but given the conditions and subsequent email discussions, most were unlikely to be attending outpatient clinics, and so could not have been recruited for this study at outpatient clinics.

### Cost per practice

The total cost of practice and patient recruitment was £4246, comprising £153 of fixed costs (designing and preparing leaflets and posters, creating and updating practice tables, meeting with practice nurses) plus £4093 of variable costs (46 practices with mean (variable) cost per practice of £88.98). Table 3 shows the cost per practice grouped according to the method used to recruit patients, from least to most successful. The total cost per practice of trying and failing to recruit 28 practices was £662.73. The average cost of recruiting patients at the six 'enthusiastic' practices that recruited 25/27 patients was £77 with the most successful practice being the one that undertook a special mailshot. All but one of these six practices were recruited by letter, or letter and email.

**Table 3:** Cost of patient recruitment by different types of practice participation

Practice participation	Number of practices	Cost per practice	Number of patients recruited	Cost per patient
No participation	28	£ 23.67	0	-

Leaflets only or leaflets and posters only	10	£130.87	1	£1308.69

Leaflets, posters and web link	2	£ 97.71	1	£ 195.41

Leaflets, posters, integrated mailshot	1	£173.53	2	£ 86.77

Leaflets, posters, weblink, researcher	4	£315.19	16	£ 78.80

Leaflets, posters, weblink, researcher, special mailshot	1	£491.86	7	£ 70.27

**Total**	**46**	**£ 88.98**	**27**	**£ 151.59**

### Feedback from practice managers regarding recruitment process

With the exception of the practices that responded to the initial letter, establishing contact with practice managers was often difficult requiring repeated attempts. Practice managers frequently reported feeling unable to promote the study because of their own work commitments and those of the other practice staff. As they were always very busy, getting their attention long enough to explain the project was difficult. Practice managers often felt rather isolated in their roles (for example "*... being a practice manager is the difficult role sandwiched between the GPs and practice staff*"). Many practice managers admitted to not reading the initial letter and explanation in any detail, under the impression that their involvement would be time consuming. One practice manager said "*if it's just having posters up and handing out leaflets then that's fine*", but generally there was a misconception about what they would be required to do. Several practices had had contact with the Expert Patient Programme [27,28] and that experience made them sceptical about our ability to recruit patients ("*I do have major reservations about being able to recruit patients, when we ourselves had a lot of difficulty getting patients involved with the Expert Patient Programme and were under quite a lot of pressure to do so*").

In another practice, a practice nurse after hearing about the project in a practice nurse meeting had requested information be resent to the practice but the practice manager response was "*thank you for supplying this information. However, I have discussed it with the doctors, who feel that we just do not have the capacity to take on anything else at this time*".

We attempted to engage more with practice managers by requesting to attend their local practice manager meeting but were told that they had *"put an email round and there was little response"*.

**Figure 2:**
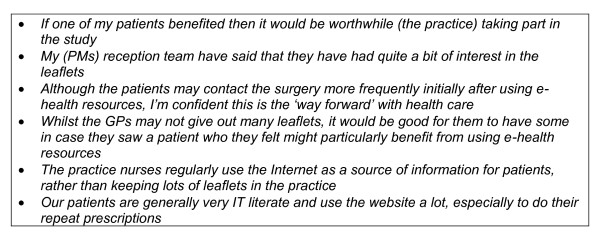
**Examples of comments from practice managers who were positive about patients' use of the Internet**.

**Figure 3:**
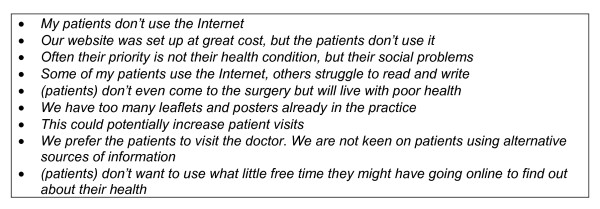
**Examples of comments from practice managers who were sceptical of their patients using the Internet, or not keen on the project**.

Success in getting practices to collaborate was determined by whether practice managers thought trying to help 'their' patients use the Internet for health would be worthwhile. While some were in favour (Figure 2) many were sceptical (Figure 3).

### Distribution of leaflets by researchers and patients' comments

To try to improve the recruitment rate and to explore why recruitment was so slow, two research assistants made 10 visits to five practices over four weeks, to distribute leaflets and to hear what patients had to say about the project. In total, 197 people were approached, with 134 (68%) taking a leaflet and 63 (32%) declining. Of the 134 who took a leaflet we recruited two people (that is, 1% of those approached).

Some patients did not wish to use computers or engage in e-health. For example, some elderly patients felt that they were *'too old to start learning now' *and did not have access to the Internet anyway. Others and had positive views about using the Internet seemed eligible for the study. Some were positive, for example, one patient with two young babies had found the Internet *'very helpful for searching health information*' with respect to her children. A late middle-aged woman spent 3 hours a day using her computer, but she only used the Internet to check emails. Some had mixed views, for example, a man said '.. *< online resources > would be really beneficial as peer support rather than 'top down' help, i.e. online discussion forums for carers, family and people with similar conditions rather than professional advice'*, but also said that some people may think the Internet is dangerous as some websites try to sell pills and things like that. Another man said that it was through his use of the Internet that he had become increasingly worried and anxious about his own health and that this had prompted him to visit his GP. However, ultimately only two out of 134 consented.

## Discussion

### Addressing e-health inequalities

Various barriers including lack of physical access, experience, attitudes, confidence or self-efficacy, knowledge, and social help may contribute to e-health inequalities [7,29,30]. Tackling e-health inequalities might address physical access through the provision of broadband (for example, the roll out of superfast broadband to rural Cornwall http://www.superfastcornwall.org/), or the provision and recommendation of e-health services, for example, in England there is marked variation of opportunities between different GPs [31] and different levels of referral by geographical area [23]. Economic factors, particularly at a time of recession are also important but are mainly solved at a regional, national or global level. The focus in medical informatics literature over recent years has been on e-health literacy [32]. But alongside e-health literacy we need to consider the support that is available from family, friends, or perhaps agencies such as Age UK, or in this case through University-run projects.

We had previously piloted email support for people recruited in outpatients, to use the Internet for health [17]. The intervention seemed to offer some potential, but key issues included when in the 'patient's journey' to recruit, and how to recruit. Participants in our previous study had suggested that support in using the Internet would have been more useful earlier. In addition, we thought that many e-health opportunities for health promotion were more relevant to primary care. We therefore wanted to explore the feasibility and cost of recruiting those people who had access to the Internet, who saw the possibility of using it for a long term condition or lifestyle change, but who could benefit from email support.

### Raising awareness in primary and secondary care for online recruitment

Online recruitment requires that potential participants are made aware of the study and can access the recruitment website. In our previous hospital study [17], participants suggested that having help to use the Internet would have been useful earlier in their condition. In this primary care study, we also recruited more people thinking of lifestyle changes or with mental health problems than in our hospital study. However, overall we recruited very few patients. The cost of recruitment was high, partly because considerable time was spent recruiting practices and agreeing what methods could be used. Although we recruited 18 practices, five 'enthusiastic' practices were responsible for 23/27 patients. These practices were easier and less costly to recruit than other practices.

In our previous hospital study, we recruited 29 participants over five weeks using leaflets in outpatient waiting rooms. We approached 864 people of which 29 (3.4%) consented via the study website. In this primary care study, 134 patients were approached by research assistants of which two (1%) consented. However, it may be easier to find and approach patients in outpatient waiting areas where there are often many patients with similar long term conditions waiting, compared to the few patients in different general practice waiting rooms. A detailed diary was not kept for our hospital study but, retrospectively, we estimated that a maximum of 70 hours were spent in recruitment and with other costs represent a total cost of £1500, i.e. about £38 per person recruited compared to, at best, £70-80 in this study.

### Recruiting patients to studies in general practice

Recruitment in general practice has often been difficult [11,12,20,33] and this study was no exception. In primary care, researchers have to negotiate access and to recruit 'at arm's length' in dispersed health centres. Practice managers act as gatekeepers to research and their views of particular research studies may be crucial. In this study, some practice manager comments showed that many were not convinced of the benefit of the proposed intervention. Unless practices are really receptive to the purpose of the research it is unlikely to 'work' [19]. On the other hand, we recruited practices and patients in deprived areas, while other managers in more affluent areas were sceptical, suggesting practice manager views may not reflect the potential of patients to benefit. So, just working with more enthusiastic practices would not have resulted in a biased sample of patients.

Although in some countries identification of patients in terms of their eligibility for research is only permitted after explicit consent from patients is obtained, in the United Kingdom, provided the appropriate controls on who sees what data are in place, recruiting patients identified via GP computer systems is possible and has been used before [19,20]. Some have reported problems in raising awareness 'offline' in general practice for online recruitment. Woodford et al. aimed to recruit patients with depression by identifying patients from GP practice registers, sending an invitation pack via post, and inviting expression of interest on a webpage [20]. Although they only recruited seven people from eleven practices the reasons for failure included the study design (lack of equipoise) and poor coding of depression in GP records. On the other hand Kuyken et al. [22,34,35] were more successful using a similar method. They claimed [19] that getting the GPs to cooperate required them to work only in primary care settings eager to develop and support a research ethos, i.e. those practices who had a 'readiness to engage'. However, that they used 'assertive outreach' (in which a researcher contacted all of those sent the initial letter unless they opted out) may explain why more of the people initially identified agreed to participate. Such personal contact requires that researchers have full access to personal information on all eligible patients, something that not all UK ethical committees would agree to. Our study aimed to recruit anonymous participants.

The main practical and cost issue in deciding whether to recruit in primary or secondary care is the number and nature of professional 'gatekeepers'. In our hospital study we were able to contact patients with permission from one 'authority'.

### Practical issues in recruiting patients in general practice

Most participating practices gave us access to use waiting room posters and leaflets as being non-intrusive on the work of the practice. However, our study has shown, that these 'passive' methods, posters and leaflets on their own, are not effective in recruitment. The most successful patient recruitment was in those practices which were easier to recruit for collaboration. There is no obvious reason why patients recruited from 'enthusiastic' practices for an e-health intervention should be different from other patients denied that possibility by practice staff. The 'enthusiastic' practices in this study came from across the city including both affluent and deprived areas. As suggested by White et al. [19], it would have been more cost effective to work only with those practices prepared to take a more pro-active approach to patient recruitment and it seems likely that this would not result in a biased sample of patients.

In our study the special mailshot was reasonably cost effective, recruiting 6 people (3% compared to less than half a percent recruited by mailshot in Woodford's study [20]). The integrated mailshot was not particularly effective, probably because of lack of incentive/enthusiasm from the practice, and the prominence of the message.

Although we suggested it to all 18 practices, only seven were able to put a website link to the study. Many of the other 11 saw it as being a difficult or time consuming task. Given that many of the practices were using the same software, or same web developer, this is probably associated with the IT literacy of the practice manager

### Strategies for recruiting e-health novices

Recruitment of patients to studies in primary care is more difficult when the study is about communication or information seeking using the Internet, and as in this study, where recruitment of one specific group on the spectrum of 'e-health readiness' is required. Our target population for this type of e-health support was people who have physical access to the Internet and sufficient Internet skills that they could deal with a website registration and email, but were not confident users of the Internet. We do not know how many people are in that category. A quarter of people in Britain have not used the Internet and many of those are not interested in going online [36]. This group was represented by some patients we contacted. But amongst those with Internet connections a proportion may benefit from support. Just over one third (36%) of Internet users say that they look up health information online, rising to 41% of Internet users aged 45-54. Overall levels of confidence among Internet users is high (87%) but drops to 73% for those aged 65 or over [36]. The ultimate aim of this research was to offer email support in using the Internet for health. Raising awareness online to recruit online, shown to be effective in many situations, remains a possibility but may not attract Internet novices. Some form of direct face to face contact, or raising awareness via more traditional media may be more appropriate.

If National Health Service centred methods of raising awareness are to be used then the best combination of methods for this target population might be used. Given the density of people with long term conditions, patients with long term conditions might be recruited in hospital outpatient areas. People with mild to moderate mental health problems, or aiming to change to healthier lifestyles, might be recruited in primary care. However, it would be most cost effective just to use practices that are willing to implement a combination of methods including special mailshot and practice nurse recruitment.

### Alternative ways of raising awareness for online recruitment

An alternative to raising awareness to patients via health services for online recruitment is to raise awareness directly to populations, either online or via the mass media. Knowing the cost-effectiveness of all methods is important to be able to decide on the best strategy. Researchers have used various media and methods to raise awareness for online recruitment, for example, Gordon et al. [14], in a study of a website supporting users of smokeless tobacco, used (a) thematic promotional releases to print and broadcast media, (b) Google ads, (c) placement of links on other Web sites, (d) limited purchase of paid advertising, (e) direct mailings to smokeless tobacco users, and (f) targeted mailings to health care and tobacco control professionals. Our own study of online advertising to recruit people to a website leading to online cognitive behavioural therapy [23], found costs per person clicking on the advert and following through to the onward link of about £1/person.

These studies show that online advertising can be an effective and inexpensive method of raising awareness of online interventions but the characteristics of those recruited need to be understood. Online recruitment may recruit those who are difficult to reach by traditional means, for example, Graham et al. [15] recruited a higher percentage of males, young adults, racial/ethnic minorities, those with a high school education or less, and dependent smokers compared to traditional methods. However, these may not be naïve users of the Internet who may benefit from e-health support. Online methods for e-health support may be worth exploring but cost-effective 'offline' methods are also needed.

### Limitations

This study was suggested by the findings of our previous outpatient based study. One possible confounder of our interpretation of our findings is that our previous hospital study was in a more affluent area [37]. It may be that more patients had access to the Internet and were at a stage of Internet use where they were willing to be helped. However, there is no information about the e-health readiness of these two populations to know if this was the case. Furthermore this pilot study used a convenience sample of only one primary care trust, the practices and practice managers may not be typical of other parts of the United Kingdom. Given that the University research team was based in the same area it may be that our results were too optimistic. We estimated costs based on marginal time costs of researchers rather than include estate and other costs (mainly because this was a marginally costed project); this again means that our results were too optimistic. The results cannot easily be applied to other countries as they are fairly dependent on the way that primary care services are organised in the United Kingdom.

## Conclusion

Recruitment to offer email support for e-health is likely to be more cost-effective in secondary care but this is less likely to recruit those with mild to moderate mental health problems or those seeking lifestyle changes. Such patients can be recruited by general practice but should be recruited via those practices that are ready to engage in a 'package' of recruitment methods including mailshots to selected patients. Leaflets and posters on their own are not effective and use of these methods may therefore result in a waste of resources. Although practices may deny their patients the opportunity to take part in such studies there is no reason to believe that the patients recruited from more enthusiastic practices are unrepresentative of the total population. Comparison between recruitment via general practice and direct to population methods via the mass media would be worthwhile.

## Competing interests

The authors declare that they have no competing interests.

## Authors' contributions

RJ had the idea for the study, managed the project, analysed the data, wrote the paper; AO collected most of the data and was responsible for day to day management of the project, helped with the analysis, and edited the paper; JB provided the e-health support intervention, provided other support and some data, edited the paper; NP had input into the design of the study, helped in management of the project, edited the paper; HS had input into the design and management of the project, reviewed methods and data collection, edited the paper. All authors read and approved the final manuscript.

## Pre-publication history

The pre-publication history for this paper can be accessed here:

http://www.biomedcentral.com/1472-6947/12/25/prepub
